# Double and multiple knockout simulations for genome-scale metabolic network reconstructions

**DOI:** 10.1186/s13015-014-0028-y

**Published:** 2015-01-09

**Authors:** Yaron AB Goldstein, Alexander Bockmayr

**Affiliations:** FB Mathematik und Informatik, Freie Universität Berlin, Arnimallee 6, Berlin, 14195 Germany

**Keywords:** Constraint-based modeling, Metabolic network, Flux coupling analysis, Reaction knockout, Gene knockout

## Abstract

**Background:**

Constraint-based modeling of genome-scale metabolic network reconstructions has become a widely used approach in computational biology. Flux coupling analysis is a constraint-based method that analyses the impact of single reaction knockouts on other reactions in the network.

**Results:**

We present an extension of flux coupling analysis for double and multiple gene or reaction knockouts, and develop corresponding algorithms for an *in silico* simulation. To evaluate our method, we perform a full single and double knockout analysis on a selection of genome-scale metabolic network reconstructions and compare the results.

**Software:**

A prototype implementation of double knockout simulation is available at http://hoverboard.io/L4FC.

## Background

Constraint-based modeling has become a widely used approach for the analysis of genome-scale reconstructions of metabolic networks [1]. Given a set of metabolites  and a set of reactions , the metabolic network is represented by its stoichiometric matrix $S \in \mathbb {R}^{\mathcal {M} \times \mathcal {R}}$, and a subset of irreversible reactions $\text {Irr} \subseteq \mathcal {R}$. The flux cone $C = \{v \in \mathbb {R}^{\mathcal {R}} \mid Sv = 0, v_{r} \geq 0, r \in \text {Irr}\}$ contains all steady-state flux vectors satisfying the stoichiometric and thermodynamic irreversibility constraints. Based on this flux cone, many analysis methods have been proposed over the years (see e.g. [2] for an overview). *Flux Balance Analysis* (FBA) [3,4] solves a linear program (LP) max{*z*(*v*)∣*Sv*=0,*l*≤*v*≤*u*} over the (truncated) flux cone in order to predict how efficiently an organism can realize a certain biological objective, represented by the linear objective function *z*(*v*). For example, one may compute the maximal biomass production rate under some specific growth conditions. *Flux Coupling Analysis* (FCA) [5,6] studies dependencies between reactions. Here the question is whether or not for all steady-state flux vectors *v*∈*C*, zero flux *v*_*r*_=0 through some reaction *r* implies zero flux *v*_*s*_=0, for some other reaction *s*.

Knockout analysis has become an important technique for the study of metabolic networks and in metabolic engineering. Starting from flux balance analysis (FBA), various *in silico* screening methods for genetic modifications have been developed, see [7,8] for an overview. On the one hand, complete methods have been proposed, which systematically explore all possible knockout sets up to a given size, e.g. [9,10]. On the other hand, there exist heuristic algorithms such as [11-14], which may be considerably faster, but in general are not complete. Klamt et al. [15-17] developed the related concept of *minimal cut sets*, which are (inclusion-wise) minimal sets of reactions whose knockout will block certain undesired flux distributions while maintaining others.

Recent progress in the development of algorithms for flux coupling analysis (FCA) [6,18] may lead to a different approach. FCA [5] describes the impact of each possible single reaction knockout in a metabolic network. It analyzes which other reactions become blocked after removing one reaction (“directional coupling”), and which reactions are always active together (“partial coupling”). As we will see, using flux coupling information inside a double or multiple knockout simulation may significantly reduce the search space, without loosing any information.

In this paper, we present an algorithmic framework for double and multiple knockouts in qualitative models of metabolic networks. We will use a lattice-theoretic approach [18], which includes classical constraint-based models at steady-state as a special case, but which is much more general. We illustrate and evaluate our method by computing full double knockout simulations on a selection of genome-scale metabolic network reconstructions. In particular, we compare the impact of single vs. double reaction knockouts on the other reactions in the network. We also show how our method can be extended to gene (in contrast to reaction) knockouts, and provide computational results for both cases.

Our algorithms are based on an efficient search for the maximal element in suitably defined lattices [18]. To simulate all double or multiple reaction knockouts, we describe a method to select a subset of the reactions as representatives for the whole system. More precisely, we partition the reaction set in equivalence classes of *partially coupled* reactions. This enables us to obtain the information about all possible double or multiple reaction knockouts much faster and to store the results in a compact format.

The approach developed in this paper is a qualitative method. We do not measure the quantitative impact of knockout sets on the cellular growth rate (or other metabolic fluxes) as this would be done in an FBA approach. Instead, we count how many reactions become blocked by a knockout, similar to the *flux balance impact degree* introduced in [19]. However, even though we do not apply FBA to evaluate the impact of a knockout, the idea of working with representatives for reaction classes via partial coupling could also be applied in an FBA context. Thus, studies like [20] and even MILP-based approaches like [21] might benefit from this method.

## Methods

### Reaction coupling in the context of knockout analysis

**Figure 1:**
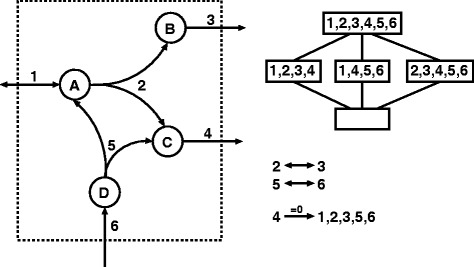
**Example network with corresponding lattice and coupling relations.** The network contains the set of metabolites $\mathcal {M} = \{A,B,C,D\}$ and the set of reactions $\mathcal {R} = \{1,2,3,4,5,6\}$. We assume that all coefficients *s*_*mr*_ of the stoichiometric matrix *S* belong to {0,+1,−1}. Thus, reaction 2 has the stoichiometry *s*_*A*2_=−1,*s*_*B*2_=*s*_*C*2_=1 and *s*_*D*2_=0. The set of irreversible reactions is $\text {Irr} = \mathcal {R} \setminus \{1\}$. A possible flux vector satisfying the steady-state condition *S* *v*=0 is *v*=(0,1,1,2,1,1) with supp *v*={2,3,4,5,6}. The corresponding lattice contains the trivial element *∅* representing the vector *v*=0 and the minimal (non-trivial) elements {1,2,3,4},{1,4,5,6} and {2,3,4,5,6}. The maximal element is {1,2,3,4,5,6}, i.e., there is no blocked reaction. a) There are two pairs of partially coupled reactions, namely 2⇔3 and 5⇔6. Therefore, no knockout sets containing reaction 3 or 5 need to be analysed. The impact of a double knockout of {3,*r*} will be the same as for {2,*r*}. b) Reaction 1 is coupled to reaction 4. Thus, a double knockout of {1,4} will have the same effect as the simple knockout of 4. In both cases, all reactions {1,2,3,4,5,6} get blocked.

We start from a metabolic network $\mathcal {N} = (\mathcal {M}, \mathcal {R}, S, \text {Irr})$ given by a set of metabolites , a set of reactions , a stoichiometric matrix $S \in \mathbb {R}^{\mathcal {M} \times \mathcal {R}}$, and a set of irreversible reactions $\text {Irr} \subseteq \mathcal {R}$, see Figure 1 for an example. The set $C = \{ v \in \mathbb {R}^{\mathcal {R}} \mid Sv = 0, v_{r} \geq 0, r \in \text {Irr}\}$ of all flux vectors $v \in \mathbb {R}^{\mathcal {R}}$ satisfying the steady-state (mass balance) constraints *Sv*=0 and the thermodynamic irreversibility constraints *v*_*r*_≥0, for all *r*∈Irr, is called the *steady-state flux cone*. A reaction $s\in \mathcal {R}$ is called *blocked* if *v*_*s*_=0, for all *v*∈*C*, otherwise *s* is *unblocked*. Two unblocked reactions *r*,*s* are called *directionally coupled* [5], written $r \stackrel {=0}{\rightarrow } s$, if for all *v*∈*C*, *v*_*r*_=0 implies *v*_*s*_=0. A possible biological interpretation is that the reactions directionally coupled to *r* are those reactions that will become blocked by knocking out the reaction *r*.

To determine which reactions are coupled, a simple approach would be to solve for each pair of reactions (*r*,*s*) two linear programs (LPs) and to check whether max {*v*_*s*_ | *v* ∈*C*,*v*_*r*_=0}= min {*v*_*s*_ | *v* ∈*C*,*v*_*r*_=0}=0. During the last years, efficient flux coupling algorithms have been developed [6,18] that drastically reduce the number of LPs to be solved, so that that genome-wide metabolic network reconstructions can now be analyzed in a few minutes on a desktop computer (compared to a couple of days of running time before).

Whether reactions are blocked or coupled does not depend on the specific flux values. It only matters whether or not *v*_*r*_=0 resp. *v*_*s*_=0. In this sense, flux coupling is a qualitative property that can be analysed by studying the set *L*^*C*^={supp *v* | *v* ∈*C*} of all *supports* of flux vectors *v*∈*C*, where $\text {supp}\; v = \{r\in \mathcal {R}\,|\,v_{r} \neq 0\}$. Each element *a*∈*L*^*C*^ is the set of active reactions of some flux vector *v*∈*C*. Therefore, we can interpret *L*^*C*^ as the set of all *possible reaction sets* or *pathways* in the flux cone *C*. Since *L*^*C*^ does not contain any information about specific flux values, we also speak of a *qualitative model* of the metabolic network .

In [18,22], we have shown that flux coupling analysis can be extended to much more general qualitative models, where the space of possible pathways $L \subseteq 2^{\mathcal {R}}$ can be any non-empty subset of the power set $2^{\mathcal {R}}$, e.g. *L*={supp *v*∣*v*∈*C*,*v* satisfies thermodynamic loop law constraints}. The definition of flux coupling needs only be slightly modified in order to be applicable to these qualitative models. A reaction $t\in \mathcal {R}$ is called *blocked in L* if and only if for all *a*∈*L*, we have *t*∉*a*. For reactions $r, s \in \mathcal {R}$ that are unblocked in *L*, we define $r \stackrel {=0}{\rightarrow } s$ in *L*, if for all *a*∈*L*, *r*∉*a* implies *s*∉*a*. To distinguish between the original flux coupling and its qualitative extension, we will call the latter *reaction coupling* from now on.

The goal of this paper is to study more general dependencies between reactions, where the flux through some reaction has to be zero, if the flux through two or more other reactions is zero.

#### **Definition1** (Joint reaction coupling).

Given a qualitative model $L \subseteq 2^{\mathcal {R}}$ of a metabolic network , let $r, s, t \in \mathcal {R}$ be unblocked reactions in *L* such that neither ${r \stackrel {=0}{\rightarrow } t}$ in *L* nor ${s \stackrel {=0}{\rightarrow } t}$ in *L* holds. We say *t* is *jointly coupled to the pair* {*r*,*s*} in *L*, written $ {\left \{ r, s \right \}}\stackrel {=0}{\rightarrow } t$ in *L*, if for all *a*∈*L*, *r*∉*a* and *s*∉*a* implies *t*∉*a*.

More generally, given a set $\mathcal {K} \subseteq \mathcal {R}$ of unblocked reactions in *L*, we say that *t* is *jointly coupled to* in *L*, written $\mathcal {K} \stackrel {=0}{\rightarrow } t$ in *L*, if for all *a*∈*L*, $a \cap \mathcal {K} = \emptyset $ implies *t*∉*a*, and $ {\mathcal {K}'} \stackrel {=0}{\rightarrow } t$ in *L* does not hold for any $\emptyset \neq \mathcal {K}' \subsetneq \mathcal {K}$.

Note that in the definition of the joint coupling ${\left \{ r, s \right \}}\stackrel {=0}{\rightarrow } t$ in *L*, we require that the simple couplings ${r \stackrel {=0}{\rightarrow }t}$ in *L* and ${s \stackrel {=0}{\rightarrow } t}$ in *L* both do *not* hold. Thus, *joint* coupling is about the synergistic effect of a pair of reactions *r*,*s* on some other reaction *t*, which cannot be obtained by either *r* or *s* alone. Similarly, $\mathcal {K} \stackrel {=0}{\rightarrow }t$ in *L* can only hold if ${\mathcal {K}'} \stackrel {=0}{\rightarrow }t$ in *L* does not hold, for any smaller knockout set $\emptyset \neq \mathcal {K}' \subsetneq \mathcal {K}$.

### Lattices and maximal elements

In [18], we presented a generic algorithm for flux coupling analysis in qualitative models. This algorithm determines the pairs of coupled reactions by computing the maximal element in suitably defined lattices.

A family of reaction sets $L \subseteq 2^{\mathcal {R}}$ is a (finite) *lattice* if *∅*∈*L* and for all *a*_1_,*a*_2_∈*L*, we have *a*_1_∪*a*_2_∈*L*. The biological interpretation of this property is that the combination of two metabolic pathways is again a pathway. In [18] we showed that *L*^*C*^ is a lattice. Any finite lattice *L* has a unique *maximal element* 1_*L*_ (w.r.t. set inclusion), which is simply the union of all lattice elements, i.e., $\displaystyle 1_{L} = \bigcup _{a \in L} a$. For any subset of reactions $\mathcal {K} \subseteq \mathcal {R}$, we may define the family

$$L_{\bot\mathcal{K}} = \left\{ a\in L \ |\ a \cap \mathcal{K} = \emptyset\right\} $$

called *Lwithout* of those reaction sets *a*∈*L* that do not contain any reaction in . If *L* is a lattice, then $L_{\bot \mathcal {K}}$ is a lattice again, and thus it has a maximal element

$$1_{L_{\bot \mathcal{K}}} = \underset{a\in L, a \cap \mathcal{K} = \emptyset}\bigcup\!\!\!\!\!\!\!a. $$

Given any lattice $L \subseteq 2^{\mathcal {R}}$, we have shown in [18] that a reaction $r \in \mathcal {R}$ is unblocked in *L* if and only if *r*∈1_*L*_. For two unblocked reactions *r*,*s*∈1_*L*_, the coupling relation $r \stackrel {=0}{\rightarrow } s$ in *L* holds if and only if $s \notin 1_{L_{\bot \{r\}}}$. In [18], we also presented an efficient algorithm to compute 1_*L*_ and $1_{L_{\bot \{r\}}}$. Once these maximal elements have been found, one can immediately determine the blocked and coupled reactions.

In this paper, we generalize these results to joint couplings. We present a method to compute the effects of double (resp. multiple) reaction knockouts based on the maximal element $1_{L_{\bot \{r,s\}}}$ (resp. $1_{L_{\bot \mathcal {K}}}$).

#### **Proposition1**.

If $L \subseteq 2^{\mathcal {R}}$is a lattice, then for any unblocked reactions *r*,*s*,*t*∈1_*L*_ we have

$$\{ r, s \} \stackrel{=0}{\rightarrow}\text{t in L if and only if } t \in \left(1_{L_{\bot\{r\}}} \cap 1_{L_{\bot\{s\}}} \right) \setminus 1_{L_{\bot\{r,s\}}}. $$

More generally, for a set of unblocked reactions $\mathcal {K} \subseteq 1_{L}$, we have

$$\mathcal{K} \stackrel{=0}{\rightarrow} \text{t in L if and only if } t \in \left(\;\underset{k \in \mathcal{K}} \bigcap1_{L_{\bot{\mathcal{K} \setminus \left\{ k \right\}}}} \right) \setminus 1_{L_{\bot \mathcal{K}}}. $$

#### *Proof*.

We prove only the first part. The second part follows by induction.

Assume $\{r, s\} \stackrel {=0}{\rightarrow } t$ in *L*. By definition, we know *t*∉*a* for all $a \in L_{\bot _{\left \{ r, s \right \}}}$, and therefore $t \notin 1_{L_{\bot {\{r, s\}}}}$. If $\{r, s\} \stackrel {=0}{\rightarrow } t$ in *L*, we also know that neither $r \stackrel {=0}{\rightarrow } t $ in *L* nor $s \stackrel {=0}{\rightarrow } t $ in *L* and that all three reactions are unblocked, i.e., *r*,*s*,*t*∈1_*L*_. As discussed in [18], we have $r \stackrel {=0}{\rightarrow } t$ in *L* if and only if $t \in 1_{L} \setminus 1_{L_{\bot \{r\}}}\phantom {\dot {i}\!}$. Since *t*∈1_*L*_, we conclude $t \in 1_{L_{\bot \{r\}}}$, and by the same argument $t \in 1_{L_{\bot \{s\}}}\phantom {\dot {i}\!}$. Hence, $ t \in \left (1_{L_{\bot \{r\}}} \cap 1_{L_{\bot \{s\}}} \right) \setminus 1_{L_{\bot \{r,s\}}}\phantom {\dot {i}\!}$.

If $ t \in \left (1_{L_{\bot \{r\}}} \cap 1_{L_{\bot \{s\}}} \right) \setminus 1_{L_{\bot \{r,s\}}}$ holds, then $t \notin 1_{L_{\bot \{r,s\}}}\phantom {\dot {i}\!}$, which implies *t*∉*a* for all *a*∈*L*_⊥{*r*,*s*}_. Since $t \in 1_{L_{\bot \{r\}}} \cap 1_{L_{\bot \{s\}}}$, we can again apply [18] to see that $r \stackrel {=0}{\rightarrow } t$ in *L* and $s \stackrel {=0}{\rightarrow } t$ in *L* do not hold. Finally, since *r*,*s*,*t*∈1_*L*_ are unblocked, we get ${\left \{ r,s \right \}} \stackrel {=0}{\rightarrow }t$ in *L*.

In [22], we considered even more general qualitative models $\emptyset \neq P \subseteq 2^{\mathcal {R}}$, where *P* needs not be a lattice. We showed there that qualitative flux coupling analysis can be done in the lattice *L*^*P*^=〈*P*〉 that is generated by *P*. The results we will present in this paper would be applicable to those qualitative models *P* as well, but for simplicity we will continue to work with models *L* that are lattices.

### Classes of partially coupled reactions

To determine joint coupling relations $\mathcal {K} \stackrel {=0}{\rightarrow } t$ in *L*, we will use as much as possible the information that can be obtained from standard couplings $r \stackrel {=0}{\rightarrow } s$ in *L*, i.e., with normal FCA. If $r \stackrel {=0}{\rightarrow } s$ in *L*, any pathway *a*∈*L* that does not use reaction *r* will also not use reaction *s*. Thus, knocking out *s* in addition to *r* will not affect the system, i.e., {*a*∈*L* | *r*,*s*∉*a*}={*a*∈*L* | *r*∉*a*}.

Additional improvements can be obtained by looking at partially coupled reactions. Two unblocked reactions *r*,*s*∈1_*L*_ are called *partially coupled* in the lattice *L*, written *r*⇔*s*, if both $r {\,\stackrel {=0}{\rightarrow }\,}s \;\text {in}\, L$ and $s {\,\stackrel {=0}{\rightarrow }\,}r \text { in } L$. The relation ⇔ is reflexive, transitive and symmetric, and thus an equivalence relation. Any equivalence relation defines a partition of its ground set into equivalence classes. In our case, $1_{L} = \bigcup _{r \in 1_{L}} \left [ r \right ]_{{\,\leftrightarrow \,}}$, where [*r*]_⇔_={*s*∈1_*L*_ | *r*⇔*s*}. An equivalence class can be represented by any of its elements, i.e., $[\!r]_{\leftrightarrow } = [\!\tilde r]_{\leftrightarrow }$ if $r \leftrightarrow {\tilde r}$. By selecting one element from each equivalence class, we get a set of *representatives* Rep⊆1_*L*_ that covers all unblocked reactions, i.e., $1_{L} = \bigcup _{r \in \texttt {Rep}} [\!r]_{\leftrightarrow }$. We will call [ *r*]_⇔_ the *coupling class* or *reaction class* of reaction *r*. Biologically, coupling classes can be interpreted as subsets of reactions that are always active together, similarly to the notion of enzyme subsets in [23].

For $r, \tilde r \in \left [ r \right ]_{\,\leftrightarrow \,}$ and *a*∈*L*, we have *r*∈*a* if and only if $\tilde r \in a$. Thus, a knockout of *r* has the same impact as a knockout of $\tilde r$. Furthermore, *r* can only be blocked by another knockout *k*∉[*r*]_⇔_ if the same holds for $\tilde r$, i.e., $k\stackrel {=0}{\rightarrow } r$ in *L* if and only if $ k \stackrel {=0}{\rightarrow }{\tilde r}$ in *L*. It follows that to analyse the effect of a knockout pair $\left \{ \tilde r,\tilde s \right \}$, one can instead knockout the corresponding representatives {*r*,*s*} with $\tilde r \in [r]_{\leftrightarrow }$ in *L* and $\tilde s \in [s]_{\leftrightarrow }$. To simulate all double knockouts, one does not have to check all pairs $\left \{ \left \{ \tilde r, \tilde s \right \} \,|\, \tilde r, \tilde s \in 1_{L}\right \}$, but it is enough to iterate over a fixed set of representatives: {{*r*,*s*}|*r*,*s*∈Rep}, see Figure 1a) for illustration. As we will see, for many genome-scale network reconstructions, there are only about half as many different equivalence classes as there are unblocked reactions (Table 1). Thus, only about 1/4 of all original pairs need to be checked. As mentioned before, although we apply this compression to reaction coupling analysis, it could also be combined with FBA-based methods.

**Table 1:** **Knockout impact on different networks**

**Model**			**Single KOs**	**Double KOs**	
	**ub**	**classes**	**Impact**	**Impact**	**ratio**
*E. coli* iJO1366	1718	1078	4.51 (16.6)	4.41 (10.1)	1.0%
*E. coli* iAF1260	1543	975	4.12 (13.7)	4.04 (9.24)	0.8%
*S. cerevisiae* iND750	631	371	5.42 (14.6)	5.52 (10.3)	2.7%
*M. tuberculosis* iNJ661	744	370	4.74 (35.6)	1.99 (5.78)	5.1%
*S. aureus* iSB619	465	207	11.7 (44.9)	7.31 (17.2)	9.2%
*H. pylori* iIT341	436	150	6.65 (58.6)	4.71 (15.5)	9.7%
*E. coli* textbook	87	55	1.96 (3.58)	15.7 (24.5)	12%

**ub:** Number of unblocked reactions in the original network.

**classes:** Number of different reaction classes, i.e., equivalence classes w.r.t. partial coupling ***⇔***.

**Single KOs impact:** Average impact of single reaction knockouts, i.e., average number of reactions classes that become blocked by a single knockout. In brackets: Average number of reactions that become blocked (belonging to different reaction classes).

**Double KOs impact**: Average *additional* impact of double reaction knockouts, i.e., average number of reactions classes that become blocked by a double knockout {*r*, *s*}, but are not blocked by a single knockout of either *r* or *s*. In brackets: Average number of additional reactions that become blocked.

**Double KOs ratio**: Percentage of pairs of (uncoupled) reaction classes that have joint coupling effects. The average numbers are determined by $\boldsymbol {\frac {1}{|K|}\sum _{\kappa \in K}\text {impact}\;(\kappa)}$ with *K* =Rep for the single, and *K* = {{*r*, *s*} | *r*, *s* ∈Rep with neither *r* $\boldsymbol {\stackrel {=0}{\rightarrow }}$ *s* in *L* nor *s* $\boldsymbol {\stackrel {=0}{\rightarrow }}$ *r* for the double knockouts.

### Algorithms

In [18], we introduced an algorithm that performs flux coupling analysis by computing maximal elements of suitably defined finite lattices $\tilde {L}$ (see also the section above on lattices and maximal elements). The basic ingredient of this algorithm is a method that checks if a given reaction $r \in \mathcal {R}$ is blocked in $\tilde {L}$, and if not returns a pathway $a\in \tilde {L}$ with *r*∈*a*. The maximal element $1_{\tilde {L}}$ of $\tilde {L}$ is computed by improving lower and upper bounds $lb,ub \in \tilde {L}$ with $lb \subseteq 1_{\tilde {L}} \subseteq ub$. In each step of the algorithm, either *lb* is increased or *ub* is decreased, until finally $lb = ub = 1_{\tilde {L}}$. The following Algorithm 1 is an extension of this method. It allows finding all the reactions in  that are unblocked after a multiple knockout $\mathcal {K} \subseteq 1_{L}$.



As discussed in [18], the flexibility of the lattice-based approach comes from hiding the search for specific pathways in a separate function FindPath. For traditional steady-state based models, FindPath can be realized by solving the linear programs $\max \{\pm v_{t}|Sv = 0, v_{\text {Irr}} \geq 0, v_{k} = 0, k \in \mathcal {K}\}$. But, one can also use other modeling hypotheses and corresponding algorithmic methods (see [22] for the example of thermodynamic loop law constraints). The skeleton of Algorithm 1 will remain the same, only the auxiliary function FindPath has to be changed.

In Algorithm 1, we perform a multiple knockout analysis with a fixed knockout set . For a full *d*-dimensional knockout analysis, we would have to iterate over all $\mathcal {K} \subseteq 1_{L}$ with $|\mathcal {K}| = d$, i.e., we would have to run the algorithm $O\left (\binom {|\mathcal {R}|}{d}\right)$ times. In each iteration, we have to solve $O(|\mathcal {R}|)$ linear programs. Since linear programming can be done in polynomial time, full *d*-dimensional knockout analysis is still polynomial (for fixed *d*), but computationally very expensive as soon as *d*>2. However, we can still use the partition of 1_*L*_ into equivalence classes of partially coupled reactions. Thus, our next Algorithm 2 calculates representatives of all jointly coupled reactions in the case of double knockouts.



In Algorithm 2, we iterate over a subset of all possible double knockouts without loosing any information. For this, we filter redundant knockout pairs such as $r \stackrel {=0}{\rightarrow } s$ in *L* (by checking $s \in 1_{L_{\bot \{r\}}}$). It is unnecessary to test such a pair, because a knockout of {*r*,*s*} is equivalent to the single knockout of *r*, see Figure 1b) for illustration. For higher-dimensional knockout sets one can proceed in a similar fashion:

Let $\mathcal {K} = \left \{ k_{1}, \ldots, k_{d} \right \} \subseteq \texttt {Rep}$ be a *d*-dimensional knockout set. Then we do *not* need to test , if any of the following conditions is fulfilled:

- $k_{i} {\,\stackrel {=0}{\rightarrow }\,}k_{j} \text { in } L$ for two reactions $k_{i}, k_{j} \in \mathcal {K}$,
- $\left \{ k_{i_{1}}, k_{i_{2}} \right \} {\,\stackrel {=0}{\rightarrow }\,}k_{j} \text { in } L$ for three reactions $k_{i_{1}}, k_{i_{2}}, k_{j} \in \mathcal {K}$,
- $\left \{ k_{i_{1}}, k_{i_{2}}, k_{i_{3}} \right \} {\,\stackrel {=0}{\rightarrow }\,}k_{j}\; \text {in}\; L$ for four reactions $k_{i_{1}}, k_{i_{2}}, k_{i_{3}}, k_{j} \in \mathcal {K}$,
- etc.

Standard FCA finds all pairs of reactions that are directionally coupled. This allows us to iterate in Algorithm 2 over all $\{r, s\} \in \mathcal {K}_{2, 1}$ with

$$ {\fontsize{7}{6}\mathcal{K}_{2, 1} = \left\{ \left\{ k_{1}, k_{2} \right\} \subseteq {\texttt{Rep}} \, |\, \text{not} k_{1} {\,\stackrel{=0}{\rightarrow}\,}k_{2} \text{ in } L \text{ and not } k_{2} {\,\stackrel{=0}{\rightarrow}\,}k_{1} \text{ in } L\right\}.} $$

$\mathcal {K}_{2, 1}$ contains all 2-tuples of coupling class representatives that are not coupled with respect to knockouts up to cardinality 1.

If one is interested to perform a full triple knockout analysis and joint coupling information is available, one can adapt the filtering technique and iterate over all $\{r_{1}, r_{2}, r_{3}\} \in \mathcal {K}_{3, 1}$ (or *K*_3,2_) with

$$\begin{array}{@{}rcl@{}} \mathcal{K}_{3, 1} &=& \left\{ \left\{ k_{1}, k_{2}, k_{3} \right\} \subseteq {\texttt{Rep}} \, |\, \text{not } k_{i} {\,\stackrel{=0}{\rightarrow}\,}k_{j} \text{ in } L,\right.\\ &&\left.\text{ for all } i \neq j \in \{1,2,3\} \right\},\\ \mathcal{K}_{3, 2} &=& \left\{ \left\{ k_{1}, k_{2}, k_{3} \right\} \subseteq {\texttt{Rep}} \, |\, \text{not } k_{i_{1}} {\,\stackrel{=0}{\rightarrow}\,}k_{j} \text{ in } L \right. \\ & & \text{ and not } \left\{ k_{i_{1}}, k_{i_{2}} \right\} {\,\stackrel{=0}{\rightarrow}\,}k_{j} \text{ in } L, \text{ for all pairwise} \\&&\left.\text{different } i_{1}, i_{2}, j \in \{1,2,3\} \vphantom{\left\{ k_{1}, k_{2}, k_{3} \right\} \subseteq {\texttt{Rep}} \, |\, \text{not } k_{i_{1}} {\,\stackrel{=0}{\rightarrow}\,}k_{j} \text{ in } L \text{ and not } \left\{ k_{i_{1}}, k_{i_{2}} \right\} {\,\stackrel{=0}{\rightarrow}\,}k_{j} \text{ in } L,}\right\}. \end{array} $$

$\mathcal {K}_{3, 1}$ contains all 3-tuples of coupling class representatives that are not directionally coupled, and $\mathcal {K}_{3, 2}$ all triples that do not contain reactions that are coupled with respect to knockouts up to cardinality 2. Similarly one could define $\mathcal {K}_{d, m}$.

While these techniques are applied here only to reaction coupling analysis, they could also be combined with FBA-based methods. Thus, if one is interested to measure the impact of all possible triple knockouts on FBA, it would be sufficient to solve $\max \left \{v_{\textit {biomass}}| Sv = 0, v_{\text {Irr}} \geq 0, v_{\mathcal {K}} = 0\right \}$ for all $\mathcal {K} \in \mathcal {K}_{3, 1}$ (if only FCA data is available) or all $\mathcal {K} \in \mathcal {K}_{3, 2}$ (if FCA and joint coupling data is available).

### The case of gene knockouts

Often metabolic networks contain regulatory rules for the gene products that catalyze the reactions, e.g. reaction *r*_1_ is catalyzed by the product of a gene *g*_1_ and reaction *r*_2_ is catalyzed by the gene product of *g*_1_ or *g*_2_. Here *r*_1_ is only possible if *g*_1_ is active, and *r*_2_ can only be blocked by a simultaneous knockout of the two genes *g*_1_ and *g*_2_. Typically, there is no 1-1 relationship between the set of genes  and the set of reactions . On the one hand, there are reactions that only get blocked by a combination of two or more gene knockouts, as indicated above in *r*_2_≡*g*_1_∨*g*_2_. On the other hand, the knockout of a single gene $g \in \mathcal {G}$ may block more than one reaction. For example, reactions *r*_1_ and *r*_3_ may both depend on the gene *g*_1_. Then one immediately gets that a knockout of *g*_1_ implies *v*_1_=*v*_3_=0. Let us further assume that FCA and double reaction knockout analysis have been performed, leading to $3\stackrel {=0}{\rightarrow } 4$ in *L* and $\left \{ 1, 3 \right \}\stackrel {=0}{\rightarrow } 6$ in *L*. Based on this information, we can extend the reactions that are blocked by the knockout of gene *g*_1_ to *v*_1_=*v*_3_=*v*_4_=*v*_6_=0. Thus, in this example we have 2 reactions (*r*_1_,*r*_3_) that are *associated to the geneg*_1_ based on information that is directly available in the network reconstruction, but in total 4 reactions (*r*_1_,*r*_3_,*r*_4_,*r*_6_) that are *coupled to the geneg*_1_. We formalize these notions in the following definition.

#### **Definition2** (Gene coupling).

Consider a qualitative model $L \subseteq 2^{\mathcal {R}}$ of a metabolic network  with reaction set  and gene set . Let $\alpha : 2^{\mathcal {G}} \rightarrow 2^{\mathcal {R}}, \Gamma \mapsto \mathcal {K}_{\Gamma }$ be a function defining a set of reactions $\mathcal {K}_{\Gamma }$ associated to the knockout of all genes in the set *Γ*. For an unblocked reaction *r*∈1_*L*_ and $\Gamma \subseteq \mathcal {G}$we define:

$$\Gamma\stackrel{=0}{\rightarrow} \text{r in L if and only if } r \notin 1_{L_{\bot {\mathcal{K}_{\Gamma}}}}. $$

We say that the reaction *r* is *coupled* to the gene knockout *Γ*. If *Γ*={*g*} is a single gene, we simply write $g\stackrel {=0}{\rightarrow } r$ in *L*.

Given the function $\alpha : 2^{\mathcal {G}} \rightarrow 2^{\mathcal {R}}$, we can determine the reactions coupled to the gene set *Γ* by applying Algorithm 1 to the set of associated reactions $\mathcal {K}_{\Gamma }$. Note that the definition of gene coupling slightly differs from the one of joint reaction coupling. Here, we do not exclude reactions that are already knocked out by single (or smaller set of) gene knockouts. This is to account for the possibility that, for example, a reaction *r* may be associated to a single gene knockout *g*_1_, but not to the double knockout {*g*_1_,*g*_2_} (assume *r*≡*g*_1_∨¬*g*_2_).

To simulate the impact of all single gene knockouts, one can perform an iteration over all genes $g \in \mathcal {G}$. Similarly, one can determine all double gene knockout effects by an iteration over all pairs of genes $\{g_{1}, g_{2}\} \subseteq \mathcal {G}$. However, in contrast to Algorithm 2, we cannot use gene class representatives to decrease the number of pairs that have to be analyzed.

## Results and discussion

To evaluate our method, we simulated all single and double reaction knockouts for a number of genome-scale metabolic network reconstructions from the BiGG-database [24]. The computations were done on a MacBook Air (2012), with 1.8 GHz Intel Core i5, 4GB RAM, and running Java Oracle JDK 1.7.45 under Mac OS X 10.9. To solve linear programs (LPs), we used CPLEX Version 12.6.

### Impact of double knockouts

Table 1 shows the impact of single and double reaction knockouts for the different networks. In most cases, the knockout of a single reaction class (due to the knockout of one or more of its reactions) blocks the reactions in 4 to 5 other reaction classes in average. The least robust system is *S. aureus* iSB619, where a single knockout has an average impact of almost 12 coupled reaction classes. In *S. aureus* iSB619, about 9.2% of all possible double knockouts {*r*,*s*} have *joint* coupling effects, i.e., there exist reactions $t \in \mathcal {R}$ that are blocked by the double knockout {*r*,*s*}, but not by a single knockout of *r* or *s* alone. This is a comparatively large number. For the bigger *E. coli* models iAF1260 and iJO1366, only around 1% of all double knockouts of two uncoupled reaction classes {*r*,*s*} have an impact that exceeds the effects of the corresponding two single knockouts. In *S. aureus* iSB619, double knockouts also have very strong combined effects. In addition to the reaction classes that would be knocked out by *r* or *s* alone, in average more than 7 reaction classes are coupled to a double knockout corresponding to a joint coupling $\{r, s\}\stackrel {=0}{\rightarrow } t$ in *L*. But, even for the most robust system, *M. tuberculosis* iNJ661, a double knockout (if its impact is different from the two single knockouts) in average has a combined effect of 2 additional knocked out classes resp. 5.8 reactions.

### Knockout options

In our next experiment, we take the opposite perspective (Table 2). We analyse how robust an average reaction is to single or double knockouts. More precisely, we ask the following question: Given a reaction *t*, what are the possible choices for a single reaction *r* resp. a pair of reactions {*r*,*s*} such that $r\stackrel {=0}{\rightarrow } t $ in *L* resp. $\{r, s\} \stackrel {=0}{\rightarrow }t$ in *L* holds. This perspective corresponds to a lab experiment for finding knockout targets for the reaction *t*. Here, we consider single reactions instead of reaction classes. This means that for $\{r, s\} \stackrel {=0}{\rightarrow } t$ in *L* with *r*,*s*,*t*∈Rep, we get |[*r*]|·|[*s*]| knockout options for all the |[*t*]| reactions that belong to the same reaction class as *t*.

**Table 2:** **Average number of knockout options**

**Model**	**Single KOs**	**Double KOs**
	**Options**	**Options**
*E. coli* iJO1366	35.1	143
*E. coli* iAF1260	26.4	78.0
*S. cerevisiae* iND750	25.6	106
*M. tuberculosis* iNJ661	82.7	120
*S. aureus* iSB619	65.9	245
*H. pylori* iIT341	143	126
*E. coli* textbook	6.92	132

**Single KOs options:** Average number of reactions *r* that lead as single knockouts to inactivity of a target reaction *t*: $\boldsymbol {\frac {1}{|1_{L}|} \sum _{t\in 1_{L}}\sum _{r\stackrel {=0}{\rightarrow } t\,\text {in}\, L}}$ 1.

**Double KOs options:** Average number of uncoupled reaction pairs {*r*, *s*} that lead as double knockouts to inactivity of a target reaction *t*: $\boldsymbol {\frac {1}{|1_{L}|} \sum _{t\in 1_{L}}\sum _{\{r, s\} \stackrel {=0}{\rightarrow }t\, \text {in}\,L}}$ 1.

For most of the studied networks, the average number of knockout options for a given target reaction is in the range of 25-85 single reactions and 100-150 reaction pairs. With all double knockout information at hand, one can reduce the set of all possible knockout candidates for a wet lab experiment to a small number, and additionally decide beforehand which of them have the smallest side effects.

### Impact on biomass production

To finish our discussion, we study the impact of knockouts on biomass production. To measure this, we counted the number of single and double knockouts that block the biomass reaction. Table 3 presents the results for the largest available models of the respective organisms. For two of them, more than one biomass reaction was available. In the case of *E. coli* iJO1366, we present the results for the two biomass reactions, for *S. aureus*, we selected 2 out of the 14 available reactions.

**Table 3:** **Number of knockouts for the biomass reaction in selected networks**

**Model**		**Single knockouts**		**Double knockouts**	
**reaction id**	**cl. size**	**classes**	**reactions**	**cl. pairs**	**reac. pairs**
*E. coli* iJO1366					
Ec_biomass_iJO1366_WT_53p95M	20	101	343	130	339
Ec_biomass_iJO1366_core_53p95M	1	80	288	90	268
*S. cerevisiae* iND750					
biomass_SC4_bal	26	54	156	60	142
*M. tuberculosis* iNJ661					
biomass_Mtb_9_60atp	160	64	154	48	83
*S. aureus* iSB619					
SA_biomass_1a	8	25	63	59	157
SA_biomass_5a	1	58	215	54	100
*H. pylori* iIT341					
BiomassHP_published	189	36	76	41	81

**class size**: Number of reactions in the same coupling class as the biomass reaction, i.e., number of reactions that carry flux if and only if the biomass reaction carries flux.

**Single Knockouts**: Number of different single knockouts (classes and reactions) that block the biomass reaction. Only reactions that are not partially coupled to the biomass (from a different reaction class) are counted.

**Double Knockouts**: Number of different double knockouts (class pairs and reaction pairs) that block the biomass reaction when combined. Only reactions that are not directionally coupled to the biomass are counted.

We observe that for most of the organisms, the number of single knockouts that block biomass production is very similar to the number of different double knockouts (corresponding to joint couplings) having this property, although the number of double knockout candidates is much larger (quadratic in |1_*L*_|).

### Algorithmic considerations

To perform a double knockout analysis, we first run standard flux coupling analysis (FCA) using the L4FC routine from [18]. Then we calculate the unblocked reactions for each double knockout of a pair of reaction class representatives. Table 4 presents the running times for six genome-scale network reconstructions and the central metabolism of *E. coli*. Even for our largest network, *E. coli* iJO1366 with its 2583 reactions, the complete simulation of all double reaction knockouts took less than 1h 10 min.

**Table 4:** **Runtime and number of solved LPs for double** ***reaction*** **knockouts (Algorithm 2)**

**Model**		**Step**			**Total**
		**Blocked**	**Couples**	**dko**	
*E. coli* iJO1366	LPs	1718	9943	133225	144886
	time	2.0	42.2	4016.4	1h 8min
*E. coli* iAF1260	LPs	1679	10780	52112	64571
	time	1.7	31.5	2688.2	45min 21s
*S. cerevisiae* iND750	LPs	597	3987	90664	95248
	time	0.33	6.8	397.8	6min 45s
*M. tuberculosis* iNJ661	LPs	327	3416	20647	24390
	time	0.33	5.6	177.7	3min 4s
*S. aureus* iSB619	LPs	144	3638	19477	23259
	time	0.09	2.8	43.2	46.0s
*H. pylori* iIT341	LPs	106	1812	6753	8671
	time	0.06	1.9	18.0	20.0s
*E. coli* textbook	LPs	26	341	1739	2106
	time	0.004	0.06	0.62	0.68s

The computation was done in three steps: Calculation of the blocked reactions, flux coupling analysis to determine the coupled reactions, and finally the double knockout simulations.

Times are given in seconds if not specified otherwise (numbers may not add up due to rounding errors).

Next we discuss the number of LPs we have to solve in order to obtain this additional information. For all our networks, double knockout analysis required solving 5 to 20 times as many LPs than single knockouts, i.e., classical FCA. While this seems to be a large number, it is relatively small compared to the complexity of the problem. A full double knockout simulation is comparable to iterating over all reactions *r*∈Rep, removing the reaction *r* and performing a single knockout simulation for each of the resulting subnetworks. Reusing known pathways as witnesses and including reaction coupling information as proposed in [18] allows performing |Rep| simulations with only 5 to 20 times the effort in LP solving. Table 1 shows that the median value for |Rep| is 370 for our networks.

In order to evaluate the runtime effect of our algorithmic improvements, we considered two variants of Algorithm 2: **Variant A (no representatives)** In the main loop of Algorithm 2, we do not iterate over all representatives *r*,*s*∈Rep,*r*<*s*, but over all pairs of uncoupled reactions *r*,*s*∈1_*L*_,*r*<*s*, with not $r \stackrel {=0}{\rightarrow } s\; in\; \textit {L}$ and not $s \stackrel {=0}{\rightarrow } r$ in *L*. **Variant B (no witnesses)** Same as Variant A. Additionally we do not save witnesses, thus $\mathcal {W} = \emptyset $.

These two experiments allow determining time savings due to representatives (comparing Algorithm 2 and Variant A) and time savings due to warm starts based on knowledge of existing reaction sets (comparing Variant A and B). We should emphasize here that Variant B is is still more efficient than a naive brute force algorithm. The runtime results are given in Table 5, where we stopped computations after a timeout of 6h. Table 5 shows that the efficiency of Algorithm 2 is mostly due to the re-use of (up to 10000) pathways as witnesses (factor 10 in the case of *E. coli textbook* and factor 100 for *S. aureus*). Nevertheless, iterating over the set of representatives adds another improvement of up to 50% (*S. aureus*). Since it takes a very small effort to calculate a set of representatives to profit from this additional speed-up, we highly recommend to iterate over representatives whenever possible.

**Table 5:** **Runtime of variants of Algorithm 2 for computing double reaction knockouts**

**Model**	**Algor. 2**	**Variant A**	**Variant B**
		**(no representatives)**	**(no witnesses)**
*E. coli* iJO1366	1h 8min	1h 59min	> 6h
*E. coli* iAF1260	45min 21s	1h 30min	>6h
*S. cerevisiae* iND750	6min 45s	8min 39s	> 6h
*M. tuberculosis* iNJ661	3min 4s	7min 42s	> 6h
*S. aureus* iSB619	46s	1min 59s	2h 32min
*H. pylori* iIT341	20s	58.1s	52min
*E. coli* textbook	0.68s	2.2s	23.0s

### Gene knockouts

Table 6 gives the runtimes and the number of LPs for single and double *gene* knockouts. To determine the reactions associated to a (double) gene knockout, we used the library JEval that allows fast evaluation of logical formulas given as Java strings. As expected we are confronted with longer runtimes up to almost 4h for double gene knockouts compared to < 70 min for double reaction knockouts. This is due to the fact that we need to check every single pair of genes instead of a representative selection like the one we could apply in double reaction knockout analysis. In spite of this, with the methods proposed here, a full simulation of double reaction or double gene knockouts on a genome-scale metabolic network reconstruction can still be performed in a reasonable time.

**Table 6:** **Runtime and number of solved LPs for single and double** ***gene*** **knockouts**

**Model**		**Step**	
		**gko**	**dgko**
*E. coli* iJO1366	LPs	719	263844
	time	1.2	3h 49min
*E. coli* iAF1260	LPs	516	229498
	time	8.6	2h 55min
*S. cerevisiae* iND750	LPs	1323	308145
	time	6.4	37min 36s
*M. tuberculosis* iNJ661	LPs	175	77346
	time	1.2	15min 59s
*S. aureus* iSB619	LPs	49	38689
	time	0.68	9min 42s
*H. pylori* iIT341	LPs	27	19348
	time	0.24	1min 52s
*E. coli* textbook	LPs	2	2023
	time	0.04	4.4s

Times are given in seconds if not specified otherwise.

## Conclusions

On the algorithmic side, this study presented the following main results:

- Algorithm 2 is an effective method for a complete double knockout analysis in genome-scale metabolic networks.
- Using Algorithm 1, it is possible to compute the impact of specific multiple knockout sets containing 3 or more reactions.
- By exploiting the information present in reaction coupling data (obtained by FCA), one can significantly decrease the number of candidates that need to be tested in double and multiple knockout simulations.

Regarding the biological data, we can make the following observations based on our computational experiments:

- In the genome-scale metabolic network reconstructions that were considered in this study, 1-10% of the possible double knockout sets have joint coupling effects. Thus, given a randomly chosen reaction pair, the probability is high that the combined effect of the double knockout (in terms of other blocked reactions) will be the same as for the two corresponding single knockouts.
- However, in all these networks, there exists a small number of double knockouts showing synergistic effects, blocking 5 to 20 additional reactions in average. These double knockouts cannot be predicted from the single knockout/reaction coupling data alone.

Due to the algorithmic improvements, we are now able to perform full double gene or reaction knockout simulations in a few hours of computation time. Thus, whenever one is interested in understanding the robustness of a network to knockouts, one should take the opportunity and run such an *in silico* simulation, before starting other more time consuming and expensive experiments.

A prototype implementation of double knockout simulation is available at http://hoverboard.io/L4FC.
